# FACILITY CHARACTERISTICS, STAFF INJURY, AND DISRUPTIVE BEHAVIOR REPORTING RATES IN VA COMMUNITY LIVING CENTERS

**DOI:** 10.1093/geroni/igz038.2353

**Published:** 2019-11-08

**Authors:** David Mohr, Kim J Curyto, Kevin W McConeghy, Jenefer M Jedele, Kelly Vance

**Affiliations:** 1 Center for Healthcare Organization and Implementation Research, VA Boston Healthcare System, Boston, Massachusetts, United States; 2 VA Western NY Healthcare System, Batavia, New York, United States; 3 Centor of Innovation in Long-Term Services and Support, Providence VA Medical Center, Providence, Rhode Island, United States; 4 Serious mental Illness Treatment Resource and Evaluation Center, Office of mental Health and Suicide Prevention, Department of Veterans Affairs, Ann Arbor, Michigan, United States; 5 Workplace Violence Prevention Program, Office of Mental Health and Suicide Prevention, Lexington, Kentucky, United States

## Abstract

Facility characteristics’ influence on staff injuries was evaluated from 2013-2018 for 105 VA CLCs. Nursing hours, nurse skill-level, resident case-mix (percent of residents with mental health or other conditions) and facility size were evaluated in a multivariable regression model. Overall the average injury rates per year were 2.7 (standard deviation 4.3) and 1.5 (2.7) in STAR-VA enrolled vs. never enrolled sites (p=0.04). Statistically significant predictors for higher staff injury rates included percent of residents with dementia, larger bed facilities, and more mental health employee coverage. Lower staff injury rates were associated with facilities with more short-stay residents. After adjustment for facility characteristics, STAR-VA sites were not an independent predictor for staff injury rates. Sites selected for enrollment in STAR-VA have higher overall injury rates which may be due to facility differences in size, staffing and proportion of residents with dementia. Implications for training and monitoring CLC sites will be discussed.

